# Difference between “Lung Age” and Real Age as a Novel Predictor of Postoperative Complications, Long-Term Survival for Patients with Esophageal Cancer after Minimally Invasive Esophagectomy

**DOI:** 10.3389/fsurg.2022.794553

**Published:** 2022-05-12

**Authors:** Zhi-Nuan Hong, Kai Weng, Zhen Chen, Kaiming Peng, Mingqiang Kang

**Affiliations:** ^1^ Department of Thoracic Surgery, Fujian Medical University Union Hospital, Fuzhou, China; ^2^ Key Laboratory of Cardio-Thoracic Surgery (Fujian Medical University), Fujian Province University, Fuzhou, China; ^3^ Key Laboratory of Ministry of Education for Gastrointestinal Cancer, Fujian Medical University, Fuzhou, China; ^4^ Fujian Key Laboratory of Tumor Microbiology, Fujian Medical University, Fuzhou, China

**Keywords:** esophagectomy, lung age, pulmonary function test, impaired pulmonary function, esophageal cancer

## Abstract

**Background:**

This study aimed to investigate whether the difference between “lung age” and real age (L–R) could be useful for the prediction of postoperative complications and long-term survival in patients with esophageal cancer followed by minimally invasive esophagectomy (MIE).

**Methods:**

This retrospective cohort study included 625 consecutive patients who had undergone MIE. “Lung age” was determined by the calculation method proposed by the Japanese Respiratory Society. According to L–R, patients were classified into three groups: group A: L–R ≦ 0 (*n *= 104), group B: 15 > L–R > 0 (*n *= 199), group C: L–R ≥ 15 (*n* = 322). Clinicopathological factors, postoperative complications evaluated by comprehensive complications index (CCI), and overall survival were compared between the groups. A CCI value >30 indicated a severe postoperative complication.

**Results:**

Male, smoking status, smoking index, chronic obstructive pulmonary disease, American Society of Anesthesiologists status, lung age, and forced expiratory volume in 1 s were associated with group classification. CCI values, postoperative hospital stays, and hospital costs were significantly different among groups. Multivariate analysis indicated that L–R, coronary heart disease, and 3-field lymphadenectomy were significant factors for predicting CCI value >30. Regarding the prediction of CCI value >30, area under the curve value was 0.61(95%: 0.56–0.67), 0.46 (95% CI, 0.40–0.54), and 0.46 (95% CI, 0.40–0.54) for L–R, Fev1, and Fev1%, respectively. Regarding overall survival, there was a significant difference between group A and group B + C (log-rank test: *p *= 0.03).

**Conclusions:**

Esophageal cancer patients with impaired pulmonary function had a higher risk of severe postoperative complications and poorer prognosis than those with normal pulmonary function. The difference between “lung age” and “real age” seems to be a novel and potential predictor of severe postoperative complications and long-term survival.

## Background

Esophageal cancer is one of the most common types of cancer and a common cause of death in Eastern countries, especially in China. The latest data from China National Cancer Center show that the incidence of esophageal cancer ranked the sixth and the mortality ranked the fourth (1, 2). Surgical resection combined with neoadjuvant chemotherapy or chemoradiotherapy is the first-line treatment for locally advanced esophageal cancer (3). However, esophagectomy is still challenging due to considerable high morbidity and mortality, requiring a long period of function recovery (4, 5).

Impaired pulmonary function, measured by pulmonary function testing (PFT), is a known risk factor for postoperative complications (6). Klevebro et al. reported a multicenter cohort study from five high-volume esophageal cancer centers in Europe and concluded that impaired pulmonary function was associated with an increased risk of postoperative complications after esophagectomy, especially for anastomotic leak and pneumonia. The preoperative PFT is necessary to facilitate treatment planning (7). Currently, the Tiffeneau–Pinelli index [forced expiratory volume in 1 s (FEV1)/forced vital capacity (FVC ratio)] >70% is considered to be acceptable pulmonary function (8). However, sometimes it is difficult to interpret PFT results to patients.

To help describe patients’ respiratory function, the Japanese Respiratory Society (JRS) proposed the concept of “lung age” (9, 10). The “lung age” has already been confirmed useful for the prediction of postoperative respiratory complications and survival in patients with lung cancer treated surgically (9). Further, “lung age” has also been proved to be associated with the occurrence, severity, and time of onset of postoperative pneumonia after esophagectomy (10).

To date, there was still no study focusing on the relationship between the difference between “lung age” and real age (L–R) and overall postoperative complications and overall survival. This study aimed to investigate whether L–R could be useful for the prediction of postoperative complications and long-term survival in patients with esophageal cancer followed by minimally invasive esophagectomy (MIE).

## Methods

### Patient Selection and Study Design

This study was approved by the Ethics Committee of Fujian Medical University, China, and followed the principles of the Helsinki Declaration. In addition, the patient’s written informed consent was obtained.

A retrospective review was conducted of 822 consecutive patients who underwent MIE (including total MIE and hybrid MIE) for esophagus cancer at Fujian Medical University Union Hospital from January 2010 to December 2017. Inclusion criteria included: aged 18–75 years old, with or without neoadjuvant following radical esophagectomy, with PFT results, with long-term follow-up results. Exclusion criteria included: with heart, lung, or liver dysfunction, or acute infection; with nonresectable tumors or metastases during exploratory surgery; and lack of preoperative PFT results. Finally, a total of 197 patients were excluded, and 625 patients were included for further analysis.

### Calculation Method of “Lung Age”

We used the calculation method of “lung age” proposed by the JRS. The calculation of “lung age” was derived from the inverse calculation of the FEV1 standard regression equation. For male: lung age (years) = [0.036*body height (cm) − 1.178 − FEV1 (L)]/0.028. For female: lung age (years) = [0.022*body height (cm) − 0.005 − FEV1 (L)]/0.0229. From the results of the PFT, we derived “lung age” in accordance with the calculation described above. According to the differences between “real age” (R) and “lung age” (L), patients were classified into three groups: group A: L–R ≦ 0 (*n *= 104), group B: 15 > L–R > 0 (*n *= 199), and group C: L–R ≥ 15 (*n* = 322).

### Concept and Calculations Method of Comprehensive Complications

Slankamenac et al. first developed Comprehensive Complications Index (CCI) in 2013 to integrate complications with their respective severity (11). CCI is a more sensitive complication index than the Clavien–Dindo (CD) classification and has been confirmed as a promising score system to evaluate the severity of complications after esophagectomy (12, 13). The CCI values range from 0 to 100; a value of 0 reflects the absence of complications, while a CCI of 100 indicates that the patient has died due to complications. CCI values above 30 indicate severe complications. Postoperative complications in the hospital were coded using the CD classification, and then we calculated the CCI on www.assessurgery.com. According to patients’ CCI values, we divided the patients into two groups: with severe complications (CCI > 30) and without severe complications (CCI ≦ 30).

### Follow-Up

All patients were followed up by outpatient or telephone at 1 month, 3 months, and 6 months after discharge, and once every 6 months in the following 3 years, and once every year thereafter until 5 years after resection. All patients underwent clinical examinations, blood tests for tumor markers, and computed tomography examinations of the neck, chest, and upper abdomen. Echocardiography and positron emission tomography examinations were performed when necessary.

### Statistical Analysis

Statistical analysis was conducted in R version 4.0.4. Continuous data with normal distribution were reported as mean ± standard deviation, otherwise were reported as median (25 quantile, 75 quantile). Categorical data were reported as *n* (%).

To compare the differences between each group, we used the chi-squared test for categorical variables and one-way ANOVA or Kruskal–Wallis test for continuous variables. Univariate analysis and multivariate analysis were performed to identify the significant predictive factors for the development of severe complications (CCI values >30) found to be relatively significant in univariate analysis using a forward stepwise selection procedure. We used area under the curve (AUC) to evaluate the prediction ability. For categorical outcomes, logistic regression models were used to calculate odds ratios (OR) with 95% confidence intervals (CI) and illustrated as a forest plot. Survival curves were estimated using the Kaplan–Meier method, and the difference in survival times among each group was calculated by the log-rank test. A two-sided *p*-values <0.05 was considered to be significant.

## Results

### Comparison of the Clinical Factors and Demographic Characteristics among Three Groups

Gender (*p *< 0.001), smoking status (*p *< 0.001), smoking index (*p *< 0.001), chronic obstructive pulmonary disease (COPD) (*p *= 0.02), American Society of Anesthesiologists (ASA) status (*p *< 0.001), lung age (*p *< 0.001), and FEV1 (*p *< 0.001) were significantly different among three groups. Real age was comparable among three groups. However, lung age increased from group A (51.26 ± 11.73) to group C (87.92 ± 14.53).

The male proportion increased from group A (59.62%) to group C (86.96%), as did smoker increased from group A (40.38%) to group C (63.35%), ASA III increased from group A (0.96%) to group C (17.78%), patients with COPD increased from group A (0.96%) to group C (10.80%). FEV1 decreased from group A (3.01 ± 0.69) to group C (2.31 ± 0.55). The distribution of clinical and demographic characteristics of the patients are given in Table 1.

**Table 1:** The clinical and demographic characteristics.

	Total (*n* = 625)	Group A (*n* = 104)	Group B (*n* = 199)	Group C (*n* = 322)	*p*
Real age	59.5 (54–64)	53 (59–63)	54 (60–64)	54 (60–64)	0.494
Lung age	75.12 ± 18.99	51.26 ± 11.73	66.83 ± 9.32	87.92 ± 14.53	<0.001
FEV1	2.54 ± 0.65	3.01 ± 0.69	2.89 ± 0.60	2.31 ± 0.55	<0.001
BMI (kg/m^2^)	21.82 (20.1–24.0)	20.5 (20.5–23.8)	20.0 (21.5–23.7)	20.1 (21.9–24.2)	0.27
Male	478 (76.48%)	62 (59.62%)	136 (68.34%)	280 (86.96%)	<0.001
ASA status					<0.001
I	150 (24%)	32 (30.77%)	46 (23.12%)	72 (22.36%)	
II	397 (65.52%)	71 (68.27%)	131 (65.83%)	195 (60.56%)	
III	78 (12.48%)	1 (0.96%)	22 (11.06%)	55 (17.08%)	
Smoking	350 (56%)	42 (40.38%)	104 (52.26%)	204 (63.35%)	<0.001
Smoking index	300 (0–800)	0 (0–600)	200 (0–800)	400 (400–800)	<0.001
Drink	88 (14.08%)	9 (8.65%)	28 (14.07%)	51 (15.84%)	0.19
Comorbidities					
COPD	54 (8.64%)	1 (0.96%)	15 (7.54%)	38 (11.80%)	0.02
Diabetes	33 (5.28%)	2 (1.92%)	15 (7.54%)	16 (4.97%)	0.11
Hypertension	119 (19.04%)	15 (14.42%)	41 (20.60%)	63 (19.57%)	0.4
Tuberculosis	21 (3.36%)	2 (1.92%)	4 (2.01%)	15 (4.66%)	0.18
CHD	13 (2.08%)	0 (0.0%)	4 (2.01%)	9 (2.79%)	0.22
Neoadjuvant chemoradiotherapy	10 (1.6%)	1 (0.96%)	5 (2.51%)	4 (1.24%)	0.45
Neoadjuvant chemotherapy	43 (6.88%)	3 (2.88%)	14 (7.04%)	26 (8.07%)	0.19
Histologic type					0.78
SCC	611 (97.76%)	103 (99.04%)	195 (97.99%)	313 (97.20%)	
Adenocarcinoma	2 (0.32%)	0 (0)	1 (0.50%))	1 (0.31%)	
Others	12 (1.92%)	1 (0.96%)	3 (1.51%)	8 (2.48%)	
Tumor location					0.12
Upper	53 (8.48%)	6 (5.77%)	23 (11.56%)	24 (7.45%)	
Middle	420 (67.2%)	71 (68.27%)	121 (60.80%)	228 (70.81%)	
Lower	152 (24.32%)	27 (25.96%)	55 (27.64%)	70 (21.74%)	
Operation type					0.82
McKeown	566 (90.56%)	91 (87.5%)	186 (93.47%)	289 (89.75%)	
Ivor Lewis	56 (8.96%)	13 (12.5%)	13 (6.53%)	30 (9.32%)	
Transhiatal	3 (0.48%)	0 (0.0%)	0 (0.0%)	3 (0.93%)	
Lymphadnectomy					0.49
2-field	563 (90.08%)	97 (93.27%)	178 (89.45%)	288 (89.44%)	
3-field	62 (9.92%)	7 (6.73%)	21 (10.55%)	34 (10.56%)	
Anastomosis site					0.32
Thoracic	574 (91.84%)	92 (88.46%)	186 (93.47%)	296 (91.93%)	
Cervical	51 (8.16%)	12 (11.54%)	13 (6.53%)	26 (8.07%)	
pT					0.41
1–2	289 (46.24%)	59 (56.73%)	90 (45.23%)	140 (43.48%)	
3–4	336 (53.76%)	45 (43.27%)	109 (54.77%)	182 (56.52%)	
pN					0.86
0–1	493 (78.89%)	81 (77.88%)	160 (80.40%)	252 (78.26%)	
2–3	132 (21.12%)	23 (22.11%)	39 (19.60%)	70 (21.74%)	
Follow-up loss	104 (16.64%)	17 (16.35%)	31 (15.58%)	52 (16.15%)	0.06
CCI index	14.54 ± 16.71	11.33 ± 14.03	13.55 ± 18.27	16.18 ± 16.34	0.004
Postoperative hospital stays (days)	14.47 ± 9.39	13.44 ± 9.94	14.17 ± 10.45	14.98 ± 8.47	0.014
Hospital stays (days)	23.47 ± 11.53	23.37 ± 14.72	22.65 ± 11.84	24.01 ± 10.08	0.021
Hospital cost	81736 ± 30795	77868 ± 23298	79686 ± 39004	84256 ± 26731	0.006

*BMI, body mass index; FEV1, forced expiratory volume in 1 s; CHD, coronary heart disease; COPD, chronic obstructive pulmonary disease; SCC, Squamous cell carcinoma; CCI, comprehensive complications index.*

### Comparison of Short-Term Results among Three Groups

**Figure 1:**
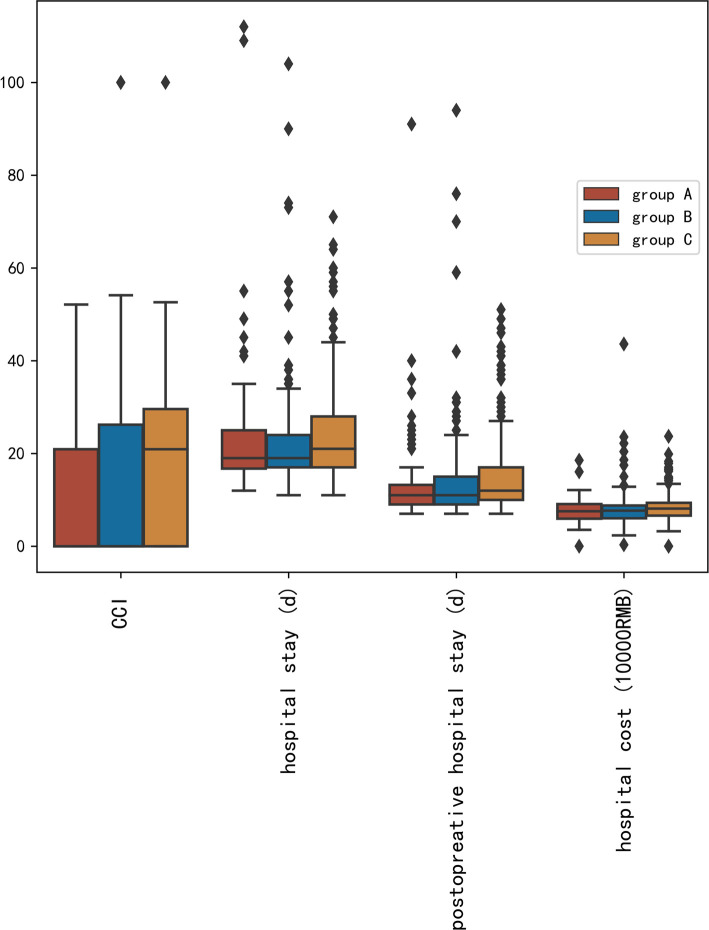
Distribution of comprehensive complications index value, hospital stay, postoperative hospital stays, and hospital cost.

The complications were scored by CCI. The CCI value increased from group A (11.33 ± 14.03) to group C (16.18 ± 16.34) (*p* = 0.004). Hospital stays, postoperative hospital stays, and hospital costs were also significantly different among the three groups (*p* < 0.05). The distribution of CCI value, hospital stay, postoperative hospital stays, and hospital cost are shown in Figure 1.

### Univariate Analysis and Multivariate Analysis of the Predictive Factors for CCI Value >30

**Figure 2:**
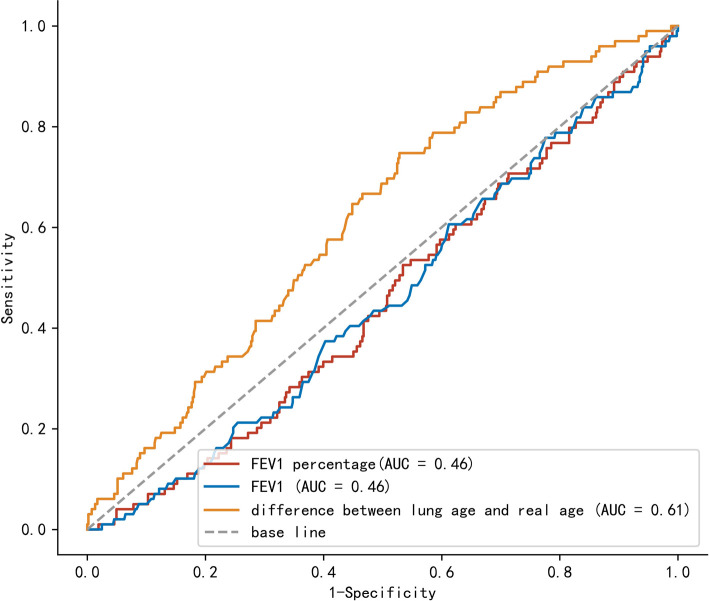
Comparison of area under the curve between lung age-real age, Fev1, and Fev1%. FeV1: forced expiratory volume in 1 s.

Univariate analysis and multivariate analysis were conducted to find the risk factors for CCI value >30, which suggested severe postoperative complications (Table 2). Gender, L–R (per 10 years), American Society of Anesthesiologists (ASA) status, coronary heart disease (CHD), lymphadenectomy field were selected for multivariate analysis. Multivariate analysis indicated that L–R (per 10 years) was a significant factor for the prediction of CCI value >30 (*p *= 0.004). The OR of L–R (per 10 years) was 1.22 (95% CI, 1.07–1.40). Regarding the prediction of CCI value >30, AUC values were 0.61 (95% CI, 0.56–0.67), 0.46 (95% CI, 0.40–0.52), and 0.46 (95% CI, 0.40–0.52), for L–R, Fev1, and Fev1%, respectively (Figure 2). After adjustment of inverse relation, the AUC value was 0.54 (95% CI, 0.48–0.60) and 0.54 (95% CI, 0.48–0.60) for Fev1 and Fev1%, respectively.

**Table 2:** Univariate analysis and multivariate analysis results of comprehensive complications index value >30.

	Univariate analysis		Multivariate analysis	
	*p*-Value	OR	95%CI	*p*-Value
Real age (<65 vs≥65)	0.22			
Gender (female vs male)	0.02	1.55	0.83–2.89	0.17
L–R (per 10 years)	0.001	1.22	1.07–1.40	0.004
FEV1	0.14			
BMI (kg/m^2^)	0.43			
ASA status (I–II vs III)	0.04	1.34	0.69–2.60	0.38
Smoking	0.22			
Drink	0.52			
Comorbidities				
COPD	0.34			
Diabetes	0.71			
Hypertension	0.75			
Tuberculosis	0.19			
CHD (without vs with)	0.01	3.71	1.06–13.08	0.04
Neoadjuvant chemoradiotherapy	0.99			
Neoadjuvant chemotherapy	0.73			
Histologic type	0.73			
Tumor location	0.06			
Operation type	0.27			
Field lymphadenectomy(2-field vs 3-field)	0.03	2.09	1.11–3.92	0.02
Anastomosis site	0.44			
pT	0.06			
pN	0.98			

*L–R, difference between lung age and real age; ASA, American Society of Anesthesiologists; CHD, coronary heart disease; COPD, chronic obstructive pulmonary disease; FEV1, forced expiratory volume in 1 s.*

### Survival Analysis

**Figure 3:**
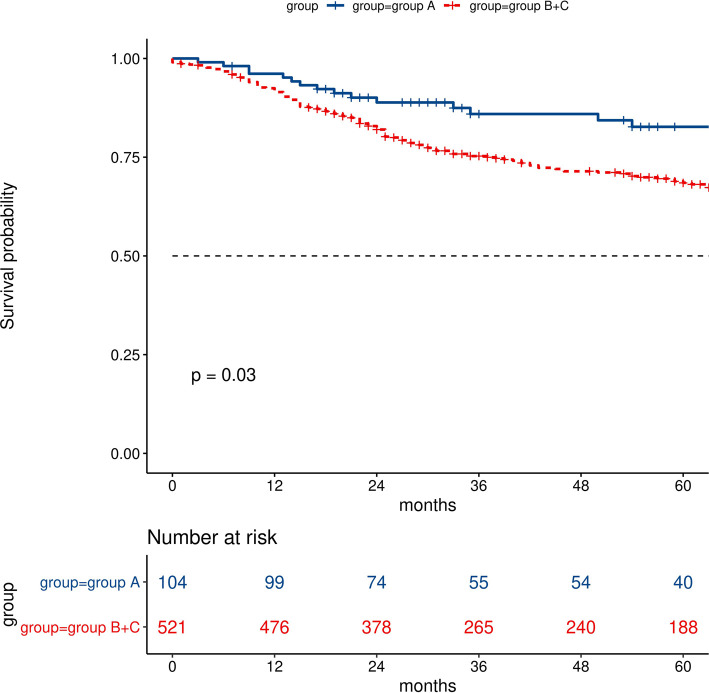
Comparisons of survival analysis between group A (difference of lung age and real age ≦0) and groups B + C (difference of lung age and real age >0).

The cumulative survival at year 3 was 85.9% in group A, 73.0% in group B, and 75.7% in group C. The overall survival in group A was significantly longer than those in group B (log-rank test: *p *= 0.026) and group C (log rank test: *p* = 0.044) However, there was no significant difference between group B and group C (log-rank test: *p* = 0.58). Further analysis showed that there was a significant difference among group A vs group B + C (log rank test: *p* = 0.03) (Figure 3).

## Discussion

This study investigated whether “lung age” could be a useful parameter for the prediction of postoperative complications and long-term survival in patients with esophageal cancer followed by MIE. There have been extensive studies focusing on MIE with poor pulmonary function (6, 14, 15). However, the PFT result may be difficult to understand. This study was the first investigation into the association between “lung age” and short-term and long-term postoperative outcomes in patients with esophageal cancer after MIE.

Our results suggested that male, COPD, ASA status was significantly associated with group classification based on the difference between lung age and real age. Furthermore, there was also an association between smoking status and index. Smoking is not only risk factor for esophageal cancer but also for COPD (16, 17). The concept of “lung age” was first proposed to motivate smoking cessation or COPD treatment. Inoue et al. reported that intensive preoperative respiratory rehabilitation could reduce the incidence of postoperative pulmonary complications in esophageal cancer patients who underwent esophagectomy (18). “Lung age” could be a parameter to explain the pulmonary function and may improve the adherence to smoking cessation before and after operation. For thoracic surgeons, lung age or the difference between lung age and real age could also help to find patients with impaired pulmonary function and give these patients preoperative respiratory rehabilitation to avoid negative events.

Through multivariate analysis, we found that L–R, CHD, 3-field lymphadenectomy were risk factors for severe postoperative complications (CCI > 30). Previous studies have confirmed that patients with impaired pulmonary function are associated with an increased risk of postoperative complications, longer hospital stay, and heavier economic burden (7, 19–22). Klevebro et al. concluded that FEV1/FVC ratio <70% was associated with an increased risk of overall postoperative complications, cardiovascular complications, atrial fibrillation, pulmonary complications, and pneumonia (7). Maruyama et al. concluded that the airflow limitation measured by FEV1% could help predict the occurrence of pneumonia after esophagectomy in patients with and without COPD (19). Merritt et al. also reported that FEV 1% < 60% is associated with major morbidity (20). Our results also confirmed that patients with impaired pulmonary function are associated with an increased risk of severe postoperative complications, more hospital cost, and longer postoperative hospital stay. L–R is a novel parameter of impaired pulmonary function. The L–R was superior to Fev1 and Fev1% in predicting major complications. After adjustment of the inverse relation, we found that the AUC value was 0.61 (95% CI, 0.56–0.67), 0.54 (95% CI, 0.48–0.60), and 0.54 (95% CI, 0.48–0.60), for L–R, Fev1, and Fev1%, respectively. Further, compared with FEV1%, FEV1/FVC, or other parameters from the PFT report, L–R is easier to be understood by patients.

Our results showed that CHD was associated with an increased risk of severe postoperative complications. This conclusion is consistent with the previous report. Klevebro et al. concluded that cardiac comorbidity is associated with an increased risk of cardiovascular and pulmonary complications, respiratory failure, and Clavien–Dindo score ≥IIIa (7). Merritt et al. concluded that preoperative coronary artery disease is a risk factor for major postoperative complications after esophagectomy following neoadjuvant chemoradiation (20). Patients with CHD are more prone to postoperative pulmonary edema due to heavy volume load. Further, patients with CHD may have a state of low cardiac output, leading to insufficient perfusion of terminal organs. This state of low cardiac output may also have adverse effects on anastomosis (20, 21).

Although many studies have compared the short-term and long-term results between 3-field lymph node dissection and 2-field lymph node dissection for esophageal cancer (18, 22–24), there is still not a consensus on whether 3-field lymph node dissection would increase the postoperative complications. A meta-analysis including 20 studies (2 randomized studies and 18 observation studies) over 7000 patients showed that 3-field lymph node dissection was associated with a higher incidence of postoperative complications, especially anastomotic leakage and recurrent nerve palsy. However, this result is limited by high heterogeneity (24). Recent PSM analysis showed that 3-field lymph node dissection resulted in more postoperative complications (25). Yamashita et al. reported that after propensity score matching (PSM) analysis 3-field lymph node dissection had similar postoperative complications with 2-field lymph node dissection (26). Our results supported that three-field lymph node dissection had a high risk of severe postoperative complications. The strength of evidence in this study was using CCI as quantitative indicators to evaluate postoperative complications.

A history of COPD is one of the most common conditions, accounting for 11.5% of newly diagnosed esophageal squamous cell carcinoma cancer patients (ESCC), 8.64% in this study. Previous reports showed that COPD is associated with a worse prognosis (27–30). Recently, Zhao et al. established a nomograms model to predict individual survival after curative esophagectomy for ESCC, and COPD is one of the independent prognostic variables (27). In this study, there was a significant difference in OS between group A (L–R ≦ 0) and group B+group C (L–R > 0) (*p *= 0.03). We contribute the OS difference to the following factors: First, immune dysfunction plays an important role in the development of impaired pulmonary function, which also promotes the rapid progression of microscopic residual disease into clinical manifestations of recurrence (29). Second, impaired pulmonary function has been found to be a risk factor for postoperative pulmonary complications. Postoperative pulmonary complications may be associated with poorer prognosis (28, 30). Thus, it may be necessary to conduct a more strict and more frequent follow-up plan for patients with L–R > 0.

The strength of this study is relatively large sample size, relatively standardized surgical procedures and perioperative management. However, this study is limited by retrospective nature and only conducted in a single institution. The majority of patients in this study were diagnosed with esophageal squamous cell carcinoma. Further, most patients underwent the trans-thoracic procedure. We tried to solve the potential selection and detection bias by strict patient selection and postoperative complication only limited in hospital stay rather than 30 days or 90 days. In order to further verify the reliability of conclusions in patients with esophageal adenocarcinoma, prospective multicenter studies are necessary.

## Conclusions

In conclusion, esophageal cancer patients with impaired pulmonary function had a higher risk of severe postoperative complications and poorer prognosis than those with normal pulmonary function. The difference between “lung age” and “real age” seems to be a novel and potential predictor of severe postoperative complications and long-term survival and has potential clinical application value.

## Data Availability

The raw data supporting the conclusions of this article will be made available by the authors, without undue reservation.
